# Migration is not the perfect answer: How the cross‐talk error correction for multiple breath nitrogen washout (MBWN_2_) parameters differs on directly collected vs. legacy data

**DOI:** 10.1002/ppul.26169

**Published:** 2022-10-04

**Authors:** Christopher Short, Mary Abkir, Sophie Pinnell, Owain Proctor, Clare J. Saunders, Jane C. Davies

**Affiliations:** ^1^ Royal Brompton and Harefield Hospitals Guys and St Thomas' Trust London UK; ^2^ National Heart and Lung Institute Imperial College London London UK; ^3^ European Cystic Fibrosis Society Lung Clearance Index Core Facility London UK

**Keywords:** biomarkers, cystic fibrosis (CF), lung clearance index (LCI), lung function testing, multiple breath washout (MBW), pulmonary function testing (PFT), pulmonary physiology

## Abstract

Recently, a cross‐talk error with commercial multiple breath nitrogen washout (MBWN_2_) software was discovered, which produced an absolute over‐reading of N_2_ of approximately 1%, i.e., 2% N_2_ read as 3%. This caused an extended tail to the washout, and over‐estimated lung clearance index (LCI_2.5_) values. Subsequently an updated and corrected software version has been released. Within the field there have been discussions on how to correct legacy data, whether to migrate or completely “rerun” raw data A‐files from the old software into the new corrected software. To our knowledge, no research has been published assessing whether either method is equivalent to *directly* collecting data in the new corrected software. We prospectively recruited 19 participants, 10 adult healthy controls and 9 people with cystic fibrosis (CF). MBWN_2_ was performed using the Exhalyzer® D first on the old 3.1.6 software and next, directly on corrected 3.3.1 software. Multiple breath washout (MBW) data directly collected in 3.3.1 was significantly different from both migrated and rerun data. A total of 7 of the 19 participants (37%; 4 CF) had a relative difference in LCI_2.5_ > 10% for both migrated and rerun data compared to 3.3.1 collected data. Our findings have implications for the Global Lung Initiative MBW project, which is accepting a combination of directly collected, A‐file reruns and migrated data to establish normative values. Further, caution must be used in clinical practice when comparing corrected legacy data versus 3.3.1 collected data for clinical interpretation. We recommend that a new baseline is collected directly on 3.3.1. before clinical interpretation and decisions are determined when comparing consecutive MBW tests.

To the editor,

Recently, Wyler et al.^1^ 2022 from the Latzin group discovered a cross‐talk error with commercial Exhalyzer D multiple breath nitrogen washout (MBWN_2_) software 3.1.6, which was in widespread use at the time. The error caused an approximately 1% absolute over‐read in N_2_, i.e., 2% N_2_ read as 3%. This caused an extended “tail” to the washout and significantly over‐estimated lung clearance index (LCI_2.5_) values. Functional residual capacity (FRC) values were also higher than those generated from body plethysmography. Since FRC derived from multiple breath washout (MBW) is a measure of the communicating lung, and FRC derived from body plethysmography is measure of the entire thoracic cavity, this is a physiological impossibility.^2^ Subsequently, the same group, worked with the software manufacturers, who released an updated and corrected software version, 3.3.1. The software manufacture instructed users that they could migrate “legacy” data collected in 3.1.6 into the new software to correct the error. The MBW central over‐reading centers (CORC's) reanalyzed legacy data using the migration method and found, whilst LCI_2.5_ values were lower and the absolute treatment effects in clinical trials smaller, the relative difference was larger, and the statistical significance of group differences ultimately unaffected.^3^

There have since, however, been discussions within the field on how to handle 3.1.6 legacy data, whether to simply migrate it or completely “rerun” raw data A‐files using the 3.3.1 algorithm. The Latzin group suggested the latter would be more accurate.^4^ However, for large clinical trials, this may simply not be feasible, and Jensen et al.^5^ reported that whilst the difference between re‐run and migrated data was significant it was very small. Despite the high interest in the cross‐sensor error correction, to our knowledge, no research has been published assessing whether either method is equivalent to *directly* collected data in the new 3.3.1 corrected software.

To address this research question, we prospectively recruited 19 participants, 10 adult healthy controls (HC: no current/previous history of respiratory disease, free of respiratory symptoms at the time of testing) and 9 people with cystic fibrosis (CF). MBW was performed within an ongoing, single center study at the Royal Brompton Hospital (clinicaltrials.gov-NCT03320382). Ethical approval was granted by South‐East Coast Research Ethics Committee (10/H1101/69). Informed written consent and age‐appropriate assent was obtained. To further assess the accuracy of migrated legacy data from 3.1.6 to create normative values, we used a large retrospective internal dataset, collected from HC adults and children. Quality control was independently performed by two European (CORC) over‐readers (CS + OP); only data accepted by both over‐readers was used.

MBWN_2_ was performed using the Exhalyzer® D (Ecomedics AG), first on the 3.1.6 software (mandated by study protocol), and next, directly on 3.3.1. Participants completing ≥2 acceptable runs on each software version within an 80‐min period are included here. 3.1.6. data were (a) migrated into 3.3.1 and (b) raw A‐files were rerun in 3.3.1. MBW runs were analysed according to the ERS/ATS consensus statement^6^ and CORC standards.^7^

Absolute and relative differences in MBW parameters were calculated between migrated, rerun and directly collected 3.3.1 data and assessed using one‐way analysis of variance with repeated measures, and post hoc Tukey's test (Prism Version 9.1; GraphPad). We performed linear regression to determine the relationship between migrated and directly collected data for the HC group and then applied the linear model to a legacy internal dataset of 203 HC (6–45 years).

MBW data directly collected in 3.3.1 was significantly different from both migrated and rerun data. In the HCs, mean (*SD*) LCI_2.5_ for the 3.3.1 collected data was 6.98 ± 0.61 compared to 6.48 (0.44) (*p* < 0.0001) for migrated and 6.52 (0.39) (*p* < 0.001) for rerun data. The CF group had mean LCI_2.5_ of 8.95 (2.92) on 3.3.1 compared to 8.16 (2.29) (*p* < 0.01) for migrated and 8.23 (2.61) (*p* < 0.01) rerun data. The mean absolute difference between directly collected and migrated or rerun data was 0.51 (0.28) [7.7 (3.8) %] and 0.48 (0.33) [7.2 (5.0) %], respectively, for HC group and 0.79 (0.66) [8.4 (5.0) %] and 0.72 (0.45) [8.5 (4.6) %], respectively, for the CF group. A total of 7 of the 19 participants (37%; 4 CF) had a relative difference >10% for both migrated and rerun data compared to 3.3.1 collected data. A lower FRC was recorded for both groups on 3.3.1 in comparison to migrated data (HC: −0.41 ± 0.21, *p* < 0.001, CF: −0.25 ± 0.24, *p* < 0.01). A lower FRC on 3.3.1 was found compared to a‐file reruns for the both groups (HC: −0.23 ± 0.18, *p* < 0.05, CF: −0.20 ± 0.25, *p* < 0.05). There was no difference found between A‐file rerun and migrated data for either LCI_2.5_ or FRC.

The upper limit of normal (ULN, 97.5%) from our legacy internal dataset on 3.1.6 was 8.30. When this dataset was migrated into 3.3.1 the new ULN was 7.41. When we applied the linear regression model (*Y* = 1.283*X‐1.329) to this dataset, the ULN became 8.10. All HC collected on 3.3.1 dataset were ≤8.10.

In conclusion, MBWN_2_ values collected in 3.1.6 and either migrated or rerun into 3.3.1 to correct for the cross‐sensor error are significantly lower than values collected *directly* in 3.3.1. We consider our findings are highly relevant to MBW field; in particular there are implications for the Global Lung Initiative MBW normative values project (https://redcap.its.dal.ca/surveys/?s=DYWF3MJR37), which is accepting a combination of directly collected, A‐file reruns and migrated data to establish normative values. We urge the investigators to consider using solely data *collected* in the 3.3.1 software to generate this normative range, understanding that this will, of course, increase the period of data collection.

Our research also has implication for clinical interpretation. Several specialist respiratory centres, on manufacturer's advice, will have migrated their legacy data into the corrected software, and will henceforth use 3.3.1 for routine MBW assessments. We found approximately 40% of values directly collected in the corrected software were >10% higher than migrated values. Recently Perrem et al 2022,^8^ demonstrated that a change in LCI_2.5_ > 10% compared to previous values should be considered clinically relevant if there was a corresponding change in FEV_1_ and/or symptoms (>15% in the absence of supportive clinical change). Therefore, in clinical practice, comparing collected versus migrated/rerun data an apparent “clinical change” may appear artefactually. The magnitude of change also appears to be greater in patients with more advanced disease (Figure 1), in whom there may be a lower threshold for treatment. We, therefore, recommend that a new baseline is collected directly on 3.3.1 before clinical interpretation and decisions are determined when comparing consecutive MBW tests. A limitation of this work is the small number of participants, and small range of LCI_2.5_ values, but considering our clear and relevant findings, we believe publicizing this reason for caution is timely.

**Figure 1:**
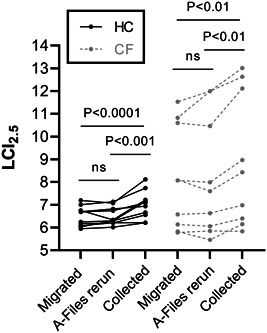
Black lines and dots represent the healthy controls (HC) whereas gray dots and lines represent people with cystic fibrosis (CF). MBW tests are a mean of at least two successful runs. One way ANOVA showed significant difference between datasets for both HC and CF groups (*p* < 0.0001 and *p* < 0.0001, respectively). Multiple comparisons between migrated and A‐file rerun data were significantly lower in both the HC and CF group in comparison to 3.3.1 directly collected data (as seen above). There we no significant differences between A‐file rerun and migrated data. ANOVA, analysis of variance.

In summary, neither the rerunning of legacy raw data nor migration of data is equivalent to collecting data directly with the cross‐sensor error corrected, 3.3.1 software. Thus, caution must be used in clinical practice when comparing migrated/rerun data vs collected data for guiding clinical decisions. With regard to clinical trials, our main recommendation is that software changes are not made mid‐study. In addition, comparisons between trials may be challenging if LCI data have been generated using different software versions. We hope to have increased awareness of this issue in investigators so that it is taken into consideration when performing power calculations and interpreting findings. Finally, all future clinical trials should be conducted using the new 3.3.1 software to correct for the cross‐sensor error.

## CONFLICTS OF INTEREST

M.A., S.P., and O.P. have no conflict of interest to declare. C.S. (Christopher Short) and C.S. (Clare Saunders) have received speaker fees from Vertex pharmaceuticals. J.C.D. has performed clinical trial leadership roles, educational and/or advisory activities for the following: Abbvie, Algipharma AS, Bayer AG, Boehringer Ingelheim Pharma GmbH & Co. KG, Eloxx, Enterprise, Galapagos NV, Genentech, ImevaX GmbH, Ionis, LifeArc, Nivalis Therapeutics, Inc., Krystal Biotech, Novartis, PARI Medical Holding GmbH, ProQR Therapeutics III B.V., Proteostasis Therapeutics INC., Pulmocide, Raptor Pharmaceuticals Inc., Recode, Vertex Pharmaceuticals.

## Data Availability

The clinical data supporting the findings of this study are not freely available as transfer agreements between institutions will be required. However, requests can be made to corresponding author.
